# How interprofessional education could benefit the future of healthcare – medical students’ perspective

**DOI:** 10.1186/s12909-020-02170-w

**Published:** 2020-07-29

**Authors:** Regwaan Imtiaz Choudhury, Muhammed Aizaz us Salam, Jai Mathur, Sharfraz Riaz Choudhury

**Affiliations:** St George’s University of London, Cranmer Terrace, Tooting, London, SW17 0RE UK

**Keywords:** Problem-based learning, National Health Service, Pre-registration, Interprofessional education, Patient safety, Patient experience, Cost-effectiveness

## Abstract

As British medical students, we believe the impact that interprofessional education can have upon the future of healthcare to be a positive one. This is if it is implemented in health professions’ pre-registration curricula worldwide. Our motivations for producing this article stem from our own experiences with IPE or rather our limited experiences during our medical school journey. We have exemplified the UK’s NHS to demonstrate how IPE would positively impact a nation’s healthcare system. With patient safety, patient experience and the economical functioning of the NHS always pertaining mainstream topics of discussion within the healthcare field, the need for improved interprofessional cohesion is now more important than ever before; especially with an increasingly demanding population. Through this article, we deeply analyse and expand upon the significance IPE has in enhancing interprofessional interactions at a pre-registration stage, in preparation for work within the NHS.

## Background

We would like to thank Lestari E et al. for their article, ‘Does PBL deliver constructive collaboration for students in interprofessional tutorial groups?’, which aptly identified the need for integrating interprofessional problem-based learning (PBL) into the curricula for pre-registration health profession programmes, in order to improve interprofessional cohesion within the clinical workspace [1]. The study demonstrated that interprofessional PBL can drive co-constructive discussions between students from different backgrounds, with a common goal of addressing patient concerns. As British medical students, we are intrigued by the potential of how interprofessional education (IPE) on a whole could further impact the future of healthcare; especially as we have not experienced adequate exposure to IPE. We take particular focus to the United Kingdom’s (UK) National Health Service (NHS) to demonstrate how interprofessional education could enhance a nation’s health system.

The NHS faces an ever-growing incidence of chronic diseases that demands multiple dimensions of care [2]. We agree that there is a distinct requirement for healthcare professionals (HCPs) to be adequately trained with cohesive skills prior to entering the NHS, as opposed to waiting for those skills to develop in-situ during clerkships. Through this article, we aim to analyse the current literature on incorporating IPE within health professional degrees in order to benefit the NHS, as well as expressing our own perspectives on the matter.

## Different forms of Interprofessional pedagogy and their advantages

PBL has yet to be formally introduced on a pre-registration interprofessional scale across the UK, where students of pharmacy, nursing, midwifery and medicine alongside other healthcare degrees, can collaboratively work through hypothetical ‘patient cases’ in groups, on a regular basis. PBL has demonstrated success across multiple studies; a systematic review directed by Qin et al. illustrated PBL’s ability to enhance critical thinking, student engagement and knowledge acquisition, compared to traditional learning methods [3]. Further research within separate healthcare degrees has demonstrated PBL’s advancements for students, with regards to their comprehension on the significance of cultivating a team-playing ethos in clinical settings [4, 5]. Such attributes acquired during intra-professional PBL sessions have inevitably led the argument for interprofessional PBL sessions to be established in UK pre-registration healthcare profession curricula, with the aim to benefit the future generations working within the NHS. Banerjee et al. discovered that medical schools failed to appropriately integrate specific attitudes to function within a Multi-Disciplinary Team (MDT) [6].

Although PBL presents many advantages, Lestari et al. acknowledge some drawbacks, such as national cultures resulting in non-medical healthcare students lacking self-confidence in sharing opinions amongst fellow medical students – this requires addressing [1]. Even though this issue predominantly lies in Asian cultures, the issue of hierarchy still exists within Western healthcare systems [7] – we are in agreement with Lestari et al. that other forms of IPE need to be delivered in aiding interprofessional PBL [1].

‘The London training ward: an innovative interprofessional learning initiative’, describes how a pilot study based on an interprofessional ward-round composed of nurses, physiotherapists and medical students, proclaimed positive signs of these different professions gaining further insight into each other’s roles [8]. Though conflict did arise, students had the opportunity to resolve this, particularly during their allocated ‘reflective’ group sessions. Not only does this allow different professions to enhance their critical thinking and debating skills, but subsides the supposed hierarchy that medical students may inherit; a ward-based environment naturally demands further input and mediation from team members due to the reality of having to deal with actual patients. Henceforth, introducing interprofessional ward-rounds into the UK pre-registration healthcare curricula, despite its logistical complexities, could well see a turn from conflict avoidance to resolution and the dissolving of unhealthy hierarchy post-qualification – both of which Lestari et al. stated were mismanaged issues during their interprofessional PBL study [1].

As the medical field undergoes constant technological advancements, we believe that medical education warrants similar renovations. Virtual IPE has been trialled and tested in the United States, whereby nursing and medical students improved their interprofessional knowledge, attitudes and team-playing attributes via co-operating with computer-generated ‘colleagues’ of different health professions [9]. The outcomes proved insignificant differences between this form of IPE and a face-to-face equivalent version of IPE. Therefore, this could open a route into further enhancing IPE for numerous pre-registration healthcare facets.

There has been difficulty in scheduling a time at which different professions, acquiring unique skills sets, can work together on a particular ‘patient case’ – as their respective courses deliver training on these attributes at different stages to one another [10]. Virtual IPE would be assisting in overcoming such problems in amalgamating IPE into relevant pre-registration courses due to its practical nature.

## Potential impact Interprofessional educational can have on the UK’s National Health Service

### Enhancing patient experience

Circumstances where interprofessional ward-round stimulation could be debated in favour of are instances where patient experience is impacted; as mentioned previously, with the increasing pervasiveness in chronic conditions amongst the UK’s population, the demand for more intricate levels of teamwork amongst health professionals is deemed necessary to keep up with the patient-centric-moving NHS. For example, a patient with Chronic Kidney Disease (CKD) commands an MDT consistent of not only a nephrologist, but a renal specialist nurse, a pharmacist, a renal dietician amongst other members. Research has in fact corroborated that acquiring an MDT for CKD patients slows the progression of CKD for some affected patients [11]; this is due to the augmented delivery of care as a result of informed decision making and evenly distributed workload during challenging times, stemming from a united MDT [12]. Consequently, this improves patient experience as they receive higher standards of care; this further emphasises the need for early exposure to interprofessional ward-round teaching for healthcare students. In theory, early exposure enables students to competently understand their peers’ roles and capacities within the NHS before working in it, whilst learning how to prevent egotistical hierarchal thinking that would hinder care.

### Improving patient safety

Addressing the incoming issues on patient safety and clinical effectiveness within the currently constrained NHS is a priority. A case highlighting how communicative flaws between health and social services serve ramifications in patient safety, were the unfortunate events which led to Baby Peter’s (Baby P) death. A Care Quality Commission report published in 2013 on the NHS’s care of Baby Peter mentioned how inadequate communication between health professionals themselves and social services resulted in a lack of urgency in the protection of Baby Peter. For example, the consultant overlooking Baby Peter’s care 2 days prior his death had failed to consult his colleagues on signs of child abuse, let alone Baby Peter’s social worker [13]. Preventable cases such as the death of Baby Peter could in fact be utilised in interprofessional PBL sessions in order that future generations of HCPs are well informed on how to co-operate in such instances and utilise critical-thinking skills.

### Cost-effectiveness

Although the NHS is under financial pressures, it remains a world leader in providing outstanding yet efficient healthcare. In order to maintain this status, the NHS will always be scouting for improved ways to be more economical. The NHS’ 10-point Efficiency Plan highlights that the management of minor cases in Accident and Emergency (A&E) requires reformation due to the wasteful referrals that prolongs waiting times in which higher-priority cases could be dealt with [14]. We are of the opinion that enhanced professional inter-personal qualities, that can be moulded through virtual IPE sessions throughout pre-registration healthcare degrees, could aid in reducing unnecessary expenditure such as those in A&E; as the crux of this issue lies between poor communication between General Practice and A&E services. Virtual IPE could enable the acquisition of inter-profession insight, such as understanding of different departmental resources - hence assisting in resolving the prodigal referrals to A&E as well as improving interprofessional communication on the back of better insight. That being said, it is important to mention that in line with achieving cost-efficiency, widespread fostering of IPE by pre-registration courses will only be one of many avenues that can be adopted, regardless of how efficient a particular IPE pedagogy may be. Other methods of being more economical within the NHS include reducing administrative costs through further implementation of digital technology for instance [15].

### Authors’ perspectives

We have a breadth of medical school experience as authors, all of us in different stages of our journey towards graduating as doctors; yet we share a mutual concern on the insufficient IPE we receive. In fact, we believe we acquire an insufficient understanding of our fellow future health professional colleagues’ roles, with our exposure to other healthcare fields often being limited to a few observational moments which arise during our clinical placements. Surely as future clinicians, should we not have a developed an understanding of the dynamics which exist within a multi-disciplinary team before graduation? Are we truly equipped to enter the unique working world of clinical medicine?

A lack of formal IPE integration into the medical pre-registration curricula means many students graduate from university and embark on their professional careers as doctors, within multi-disciplinary clinical settings, without ever sensing a deep appreciation and understanding for the roles their fellow non-physician healthcare professionals occupy. This impedes the cohesion required for an optimal work environment. Implementation of IPE sessions into numerous healthcare degrees seems to be the most efficient and practical method in order to address these issues, which is essential to further enhance the delivery of healthcare for patients and for the future progression of the NHS.

## Conclusion

Gateways for establishing IPE into the pre-registration curricula for healthcare students is materialising. The doctors’ regulatory body in the UK, The General Medical Council, in conjunction with other healthcare professions’ councils, have produced reports on their own respected degree outcomes for provisional licensing; universal goals between the professions have since derived constructing a mapping exercise [16]. This enables the groundworks from which curriculum developers can construct a strategy in which IPE can take place. Now, however, the paradigm must switch to making IPE a mainstay amongst pre-registration curricula. As Paradis E and Whitehead CR mention, the way forward for IPE would be to revise its delivery by combining pre-registrational, uniprofessional education for collaboration via building alliances across educational institutions and hospitals, on every governing level [17].

With the affairs expanded upon in this article surrounding patient experience, patient safety and cost-effectiveness in the NHS, though timely and costly, what is sown will surely reap not only long-term financial benefits but socio-economic savings for the NHS also. To conclude, we are of the opinion that more research into IPE within healthcare degrees must be conducted, in order that further answers can be deduced to aid in engineering an effective method of delivering this form of training [18]. Instilling IPE nationwide amongst degree programmes provides the opportunity for different professions to learn about each other and to learn together; in turn, making us as medical students feeling better prepared [19]. It is crucial that this topic is given more priority before it reaches ‘Accident and Emergency’.

## Data Availability

Not applicable.

## Abbreviations

A&E: Accident and Emergency
CKD: Chronic Kidney Disease
HCP: Health Care Professional
IPE: Interprofessional Education
MDT: Multi-Disciplinary Team
NHS: National Health Service
UK: United Kingdom
PBL: Problem Based Learning
